# Ellagic acid alleviates paraquat-induced oxidative stress and metabolic disturbances in the muscle of piglets

**DOI:** 10.3389/fvets.2026.1998356

**Published:** 2026-10-29

**Authors:** Yuankun Deng, Nan Wang, Yuxin Xiao, Jing Wang, Xiyuan Bao

**Affiliations:** 1 Hunan Provincial Key Laboratory for the Products Quality Regulation of Livestock and Poultry, College of Animal Science and Technology, Hunan Agricultural University, Changsha, China; 2 Yuelushan Laboratory Breeding Program, Changsha, China; 3 Department of General Surgery, No. 924 Hospital of Joint Logistics Support Force of PLA, Guilin, Guangxi Zhuang Autonomous Region, China

**Keywords:** amino acid metabolism, ellagic acid, fatty acid metabolism, muscle, oxidative stress, paraquat, piglets

## Abstract

Oxidative stress disrupts nutrient metabolism and may impair muscle development in piglets. This study investigated whether dietary ellagic acid (EA) supplementation alleviates paraquat (PQ)-induced oxidative stress and metabolic disturbances in the longissimus dorsi muscle of piglets. Piglets were assigned to a control group, a PQ-challenged group, and a PQ-challenged group supplemented with 0.01% EA. PQ challenge did not significantly affect final body weight or average daily gain but impaired antioxidant status, as evidenced by decreased serum total antioxidant capacity and glutathione peroxidase activity and reduced superoxide dismutase and glutathione levels in the longissimus dorsi muscle. EA supplementation significantly increased these four antioxidant indices relative to PQ alone. PQ also decreased the expression of several muscle development-related genes, including *MYOD, MYOG, DESMIN,* and *CAV3*, whereas EA significantly increased *CAV3, FOXO1, P21*, and *MCP-1* expression relative to PQ alone without significantly altering *MYOD, MYOG*, or *DESMIN*. Furthermore, PQ markedly altered the amino acid profile of the longissimus dorsi muscle and reduced the expression of amino acid transporters, including *SLC1A1* and *SLC6A6*. EA supplementation increased muscle Tau, Glu, Ile, Leu, Phe, and Pro concentrations, increased *SLC1A1* and *SLC6A6* expression, and decreased *PRODH, OAT*, and *ODC* expression relative to PQ alone. PQ challenge also disrupted the long-chain fatty acid profile and suppressed genes involved in fatty acid handling, whereas EA increased *FABP3, CD36,* and PLIN3 expression and increased C18:3N-3 and C22:1N-9 relative to PQ alone. Overall, these findings suggest that EA may alleviate selected PQ-associated biochemical and metabolic disturbances by improving antioxidant status and modulating muscle amino acid and lipid metabolism.

## 1 Introduction

Oxidative stress (OS) is an important physiological challenge affecting the health, growth, and metabolic homeostasis of weaned piglets. Excessive production of reactive oxygen species (ROS) can disrupt redox homeostasis, impair nutrient utilization, and cause cellular and tissue damage (1–3). Paraquat (PQ), a potent redox-cycling compound, has therefore been widely used to establish experimental models of OS (4). Previous studies have demonstrated that PQ exposure induces oxidative injury in piglets, particularly in the intestine and liver, and is accompanied by impaired antioxidant capacity and enhanced inflammatory responses (5–7). However, the effects of PQ-induced OS on skeletal muscle homeostasis and nutrient metabolism in piglets remain less well characterized.

Ellagic acid (EA) is a naturally occurring polyphenol with well-established antioxidant and anti-inflammatory properties (8, 9). Dietary EA supplementation has been reported to alleviate PQ-induced intestinal and hepatic injury in piglets by enhancing antioxidant defenses and modulating antioxidant-related signaling pathways (5, 10, 11). In addition, EA supplementation has been shown to improve antioxidant capacity and intestinal health in weaned piglets (12–15). These findings suggest that EA may exert protective effects against oxidative damage in multiple tissues. However, whether EA can alleviate PQ-induced oxidative injury and metabolic disturbances in skeletal muscle remains unclear.

The longissimus dorsi muscle is an important tissue for postnatal growth, protein deposition, and nutrient metabolism in pigs. Normal muscle development and function depend on the coordinated regulation of cellular homeostasis, nutrient availability, and metabolic processes (16–18). Amino acids are essential substrates for muscle protein synthesis and also participate in energy metabolism and other cellular functions. The composition, transport, and utilization of amino acids in skeletal muscle are therefore closely associated with muscle growth and metabolic homeostasis (19–25). Oxidative stress may disturb amino acid availability and metabolism, thereby affecting muscle development and function.

Lipid metabolism is also essential for maintaining energy supply and cellular integrity in skeletal muscle. Long-chain fatty acids serve as important energy substrates and structural components of cellular membranes, while their uptake, transport, storage, and oxidation are tightly regulated to maintain lipid homeostasis (26–29). Disruption of these processes under oxidative stress may contribute to metabolic imbalance and impair muscle function. Therefore, characterization of alterations in amino acid and lipid metabolism may provide important insights into the metabolic consequences of oxidative stress in skeletal muscle.

However, the role of EA in protecting skeletal muscle against PQ-induced oxidative and metabolic disturbances remains unclear. Therefore, this study investigated the effects of dietary EA supplementation on growth performance, antioxidant status, muscle development, and amino acid and fatty acid metabolism in the longissimus dorsi muscle of PQ-challenged piglets. We hypothesized that EA would alleviate PQ-induced oxidative stress and metabolic disturbances in piglet muscle.

## 2 Materials and methods

### 2.1 Animals and experimental design

A total of 18 healthy castrated male piglets (Duroc × Landrace × Large Yorkshire), weaned at 21 days of age, were randomly assigned to three treatment groups (*n* = 6 per group) using Microsoft Excel, with similar initial body weights across groups. The treatments were as follows: the control group (Control), fed a basal diet and intraperitoneally injected with 0.9% sterile saline; the PQ group, fed a basal diet and intraperitoneally injected with paraquat; and the EA group, fed the basal diet supplemented with 100 mg EA/kg diet and intraperitoneally injected with paraquat. The EA supplementation level was selected based on previous research findings (5). EA, with a purity of 98%, was provided by Sai’en Inc. (Xi’an, China). The experimental design is illustrated in Figure 1. Paraquat solution was prepared by dissolving paraquat powder (Sigma–Aldrich, Inc., St. Louis, MO, USA) in 0.9% sterile saline to a final concentration of 4 mg/mL. On day 18 of the experiment, piglets in the PQ and EA groups received a single intraperitoneal injection of paraquat at a dose of 4 mg paraquat ion/kg body weight, whereas piglets in the Control group received an equivalent volume of 0.9% sterile saline. The entire experimental period lasted 21 days. All piglets were individually housed in an environmentally controlled nursery with hard plastic slatted flooring and had ad libitum access to feed and water throughout the experiment. The basal diet was formulated to meet or exceed the nutrient requirements of weaned piglets recommended by the National Research Council (NRC, 2012) (Table 1). At the end of the 21-day experimental period, all piglets were humanely euthanized by intravenous administration of sodium pentobarbital at a dose of 100 mg/kg body weight, in accordance with the approved animal welfare and ethical guidelines. After confirmation of death, the animals were exsanguinated, and blood and tissue samples were collected immediately for subsequent analyses.

**Figure 1:**
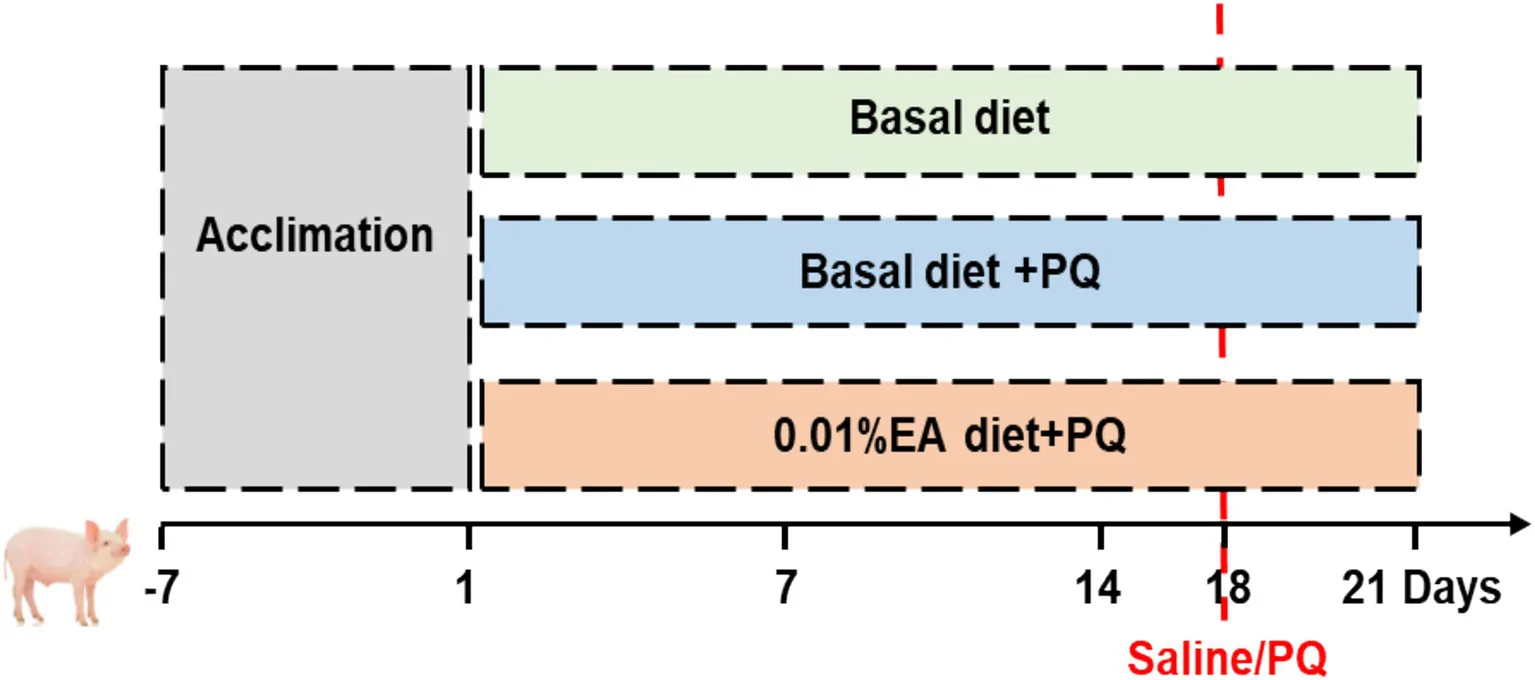
Animal experimental design.

**Table 1:** Ingredient composition of diets.

Items	Percent (%)
Corn	32.20
Extruded corn	30.00
Soybean meal	10.14
Fermented soybean	8.00
Whey powder	5.00
Fish meal	5.00
Soybean oil	2.00
Glucose	3.00
L-lysine	0.55
DL-methionine	0.07
L-threonine	0.20
L-tryptophan	0.04
Limestone	0.20
Calcium hydrophosphate	0.40
Calcium formate	0.60
Sodium chloride	0.40
Citric acid	0.80
Choline chloride	0.10
ZnO	0.20
Antioxidant	0.10
Premix^a^	1.00
Total	100
Nutritional value	
Dry matter^b^	89.58
Crude protien^b^	16.20
Ether extract^b^	17.58
DE, MJ/kg	15.41

a The premix provided the following per kg of diets: Zn (ZnO) 50.00 mg, Cu (CuSO4) 20.00 mg, Mn (MnO) 55.00 mg, Fe (FeSO4) 100.00 mg, I (KI) 1.00 mg, Co (CoSO4) 2.00 mg, Se (Na2SeO3) 0.30 mg, VA 8255.00 IU, VD3 2000.00 IU, VE 40.00 IU, VB1 2.00 mg, VB2 4.00 mg, VC 100.00 mg, VK3 1.50 mg, VB6 10.00 mg, VB12 0.05 mg, nicotinic acid 30.00 mg, folic acid 2.00 mg, biotin 0.20 mg, pantothenic acid 15.00 mg.

b Is measured value.

### 2.2 Sample collection

At the end of the experimental period, piglets were euthanized for serum and longissimus dorsi muscle sample collection. After overnight fasting, approximately 10 mL of blood was aseptically collected from the jugular vein and centrifuged at 3,000 × *g* for 10 min at 4 °C to obtain serum. The serum samples were stored at −80 °C until further analysis. Longissimus dorsi muscle samples were rapidly collected, immediately snap-frozen in liquid nitrogen, and stored at −80 °C for subsequent biochemical, gene expression, and fatty acid analyses.

### 2.3 Growth performance

Piglets were individually weighed at the start of the experiment and on days 18 and 21. Average daily gain (ADG) was calculated as body weight gain divided by the number of days in each interval.

### 2.4 Blood parameters analysis

Serum biochemical parameters were determined spectrophotometrically using an automated biochemical analyzer (BS-200; Mindray, Shenzhen, China). The measured parameters included total protein (TP), albumin (ALB), globulin (GLB), alanine aminotransferase (ALT), aspartate aminotransferase (AST), alkaline phosphatase (ALP), creatinine (CREA), glucose (GLU), triglycerides (TG), total cholesterol (TC), high-density lipoprotein cholesterol (HDL-C), and low-density lipoprotein cholesterol (LDL-C).

### 2.5 Determination of antioxidant parameters

Antioxidant parameters in serum and longissimus dorsi muscle were determined using commercial porcine enzyme-linked immunosorbent assay (ELISA) kits (Jiangsu Meimian Industrial Co., Ltd., Yancheng, Jiangsu, China) according to the manufacturer’s instructions. The parameters assessed included total antioxidant capacity (T-AOC; MM-77610O2) and glutathione peroxidase (GPx; MM-1927O2) in serum, as well as superoxide dismutase (SOD; MM-0450O2) and glutathione (GSH; MM-1915O2) in longissimus dorsi muscle.

### 2.6 Determination of amino acid contents

The free amino acid concentrations in serum and longissimus dorsi muscle were determined using an automatic amino acid analyzer (L-8900, Hitachi, Tokyo, Japan). Sample preparation was performed as follows. Briefly, longissimus dorsi muscle samples were homogenized with 5 mL of 0.01 M hydrochloric acid and subjected to ultrasonic extraction for 30 min. After centrifugation, 1 mL of the supernatant was mixed with 0.8% sulfosalicylic acid solution for protein precipitation. The mixture was incubated at 4 °C for 15 min and subsequently centrifuged at 10,000 rpm for 20 min. The resulting supernatant was filtered through a 0.22-μm membrane filter prior to HPLC analysis. Serum samples were processed using the same procedure, except that the initial homogenization and ultrasonic extraction steps were omitted.

### 2.7 Real-time quantitative PCR (RT-qPCR)

Total RNA was extracted from longissimus dorsi muscle samples using TRIzol reagent (Cat. No. 15596026; Thermo Fisher Scientific, USA) according to the manufacturer’s instructions. RNA concentration and purity were assessed using a NanoDrop One microvolume UV–Vis spectrophotometer (Thermo Fisher Scientific, USA). The A260/A280 ratios ranged from 1.8 to 2.1. Genomic DNA removal and complementary DNA (cDNA) synthesis were performed using an Evo M-MLV RT Kit with gDNA Clean for qPCR (Cat. No. AG11705; Accurate Biotechnology (Hunan) Co., Ltd., Changsha, China). Briefly, 1 μg of total RNA was subjected to the genomic DNA removal step included in the kit and subsequently reverse-transcribed in a total reaction volume of 10 μL using a combination of oligo(dT)₁₈ primers and random hexamers, according to the manufacturer’s instructions. The resulting cDNA was diluted 10-fold with nuclease-free water and used as the template for quantitative real-time PCR (qRT-PCR). qRT-PCR was performed using a SYBR Green Premix Pro Taq HS qPCR Kit (Accurate Biotechnology (Hunan) Co., Ltd., Changsha, China) on a LightCycler 480 II Real-Time PCR System (Roche). Each 10 μL reaction contained 5 μL of 2 × SYBR Green master mix, 0.4 μL each of the forward and reverse primers, 4 μL of 10-fold diluted cDNA, and 0.2 μL of nuclease-free water. The amplification conditions consisted of initial denaturation at 95 °C for 30 s, followed by 35 cycles of denaturation at 95 °C for 5 s, annealing at 60 °C for 15 s, and extension at 72 °C for 10 s. Melting-curve analysis was subsequently performed to assess amplification specificity. Each group included six biological replicates, and each sample was analyzed in three technical replicates for each gene. The mean Ct value of the technical replicates was used for subsequent analysis. Relative expression levels of target genes were calculated using the 2^−ΔΔCt^ method and normalized to β-actin expression (5, 30). The primer sequences used for qRT-PCR are listed in Table 2.

**Table 2:** Primer sequences.

Gene	Forward primer 5′–3′	Reverse primer 5′–3′
*β-Actin*	CTGCGGCATCCACGAAACT	AGGGCCGTGATCTCCTTCTG
*MYOD*	AACCCCACTTCTATGACGGG	TTATCTTCCAGGGGCACTCG
*DESMIN*	GCTGGGGGCCCTGTC	GTAAGAGGACACGCGCTGG
*FOXO1*	GCAAATCGAGTTACGGAGGC	AATGTCATTATGGGGAGGAGAGT
*SLC1A1*	GGCGCGGTGAGCAATCT	GAGCGCAGTGAAGGAAGGTT
*SLC1A2*	GGTTCTAAGGGACTGGCTTGC	ACGGGGAGAGAAAGACTCACT
*SLC6A6*	ATGGGTGATGCTGAGAGCTG	CCAGCGAGCGGAGCAG
*SLC36A1*	AGCCCAGGGAAATCTCCTCA	GCGCGTCATACACTTGCTTC
*PRODH*	AAACTTGCTACCTCCAGAGCC	CACCATCAAAGTTCGGGGGC
*OAT*	AAGAAAGGATAGTTGGGTTGCTG	GTCGCAGAAGCCACTGTAGAA
*ODC*	AGTAACGAGGAGTTGGACTGC	TTTGAGCCATCTCAGGTGCTT
*SLC25A2*	AGGCTGGCAAAAAGCCCTAA	ATCTTTTGATCCCCCGGACG
*MYOG*	CTGGTGGCAGGAACAAGTCT	GCAGGTAGTTTTCCCCGTCA
*P21*	GAGGTTTGCCTGGGGACTG	TGGAAGGAATGTGGCTGTGG
*CAV3*	CCCAGATCGTCAAGGACATTC	GTGTAGCTCACCTTCCACACG
*MCP-1*	CATCCTCCAGCATGAAGGTCT	ATTAATTGCATCTGGCTGGGC
*FABP1*	TTCATGAAGGCAGTCGGTCT	CACGATTTCCGATGTCCCCT
*FABP3*	TGGAAGCTAGTGGACAGCAAG	TTGTGGTAGGCTTGGTCATGT
*FABP4*	TCCTGAAGGTTACGGCTTCT	AAGCCCACTCCCACTTCTTT
*FATP4*	AGCCTGCTCCTTCCAAACTG	CCACTAGAGATGCCCCAAGC
*CD36*	CACTGCCTCACTGGAGTGTT	TGCTTCAAGTGCTGGGTCAA
*CAV1*	CGTAGACTCGGAGGTAGGCA	GGGCAGCAGTCGGGATATTT
*DGAT1*	CGGCAGGAGGCGGTG	CCTCTGCCTGCACCCTG
*PLIN2*	TGCTGCTGGTCGATTTCTTACT	GTGGAGCTCACCAAGGGTAG
*PLIN3*	CTTGAAGTAGCTGCTGTTGCAC	CCATAGCTCCCAGACCTTCCT
*PNPLA2*	CCCAAAGAGACGACGTGGAA	CGTAGATGTGCTTGGCGTTG
*CPT1B*	GCTGTGGCCTTCCAATTCAC	AGGATGCCATTCTTGATGCGA
*PPARα*	CCAACATGGTGGACACGGAA	GCTTCCAGAACTGTCCTCACC

### 2.8 Determination of medium and long chain fatty acids

Medium- and long-chain fatty acids in the longissimus dorsi muscle were analyzed by gas chromatography coupled with flame ionization detection (GC-FID) using an Agilent 6890N gas chromatograph (Agilent Technologies, Chicago, IL, USA) equipped with a DB-23 capillary column (60 m × 0.25 mm × 0.25 μm). Briefly, LD muscle samples were freeze-dried and ground into a fine powder. Approximately 0.1 g of each sample was accurately weighed into a 50-mL centrifuge tube. Subsequently, 4 mL of a benzene–petroleum ether mixed solvent was added, and the tubes were sealed and extracted for 24 h. After extraction, 4 mL of potassium hydroxide–methanol solution was added, and the mixture was vortexed for 3 min and allowed to stand for 30 min. Ultrapure water was then added to facilitate phase separation. The upper organic phase was collected, and an appropriate amount of anhydrous sodium sulfate was added to remove residual moisture. Finally, 200 μL of the resulting solution was diluted with 800 μL of hexane and filtered through a 0.22-μm membrane filter prior to GC-FID analysis. A 37-component fatty acid methyl ester (FAME) standard mixture (Sigma-Aldrich; 47,885-U) was used as the reference standard for GC-FID analysis.

### 2.9 Statistical analysis

All statistical analyses were performed using IBM SPSS Statistics 20.0 (IBM Corp., Armonk, NY, USA). Differences between two groups were analyzed using an independent-samples *t*-test. Specifically, comparisons were performed between the Control and PQ groups and between the PQ and EA groups. Results are presented as the mean ± standard error of the mean (SEM). Differences were considered statistically significant at *p* < 0.05. In the tables, *p*_t_-value^a^ represents the comparison between the Control and PQ groups, whereas *p*_t_-value^b^ represents the comparison between the EA and PQ groups.

## 3 Results

### 3.1 Effects of EA supplementation on growth performance in PQ-challenged piglets

As shown in Table 3, Figure 2, during the pre-challenge period (days 1–18), initial body weight (BW), BW on day 18, and average daily gain (ADG) did not differ among the control, PQ, and EA groups (*p* > 0.05). Likewise, during the PQ-challenge period (days 18–21), no between-group differences were observed in BW on day 18, final BW, or ADG (*p* > 0.05). Paired-samples *t*-tests showed that BW significantly increased from day 18 to day 21 in the control and EA groups (*p* < 0.05), whereas no significant change was observed in the PQ group (*p* > 0.05).

**Table 3:** Growth performance of piglets.

Period	Item	Control group	PQ group	EA group	*P*_t_-value^a^	*P*_t_-value^b^
1–18d	Initial BW (kg)	8.81 ± 0.45	8.79 ± 0.49	8.81 ± 0.44	0.88	0.97
	18d BW (kg)	15.34 ± 1.10	16.80 ± 1.02	16.15 ± 0.92	0.17	0.92
	ADG (g/d)	477.38 ± 88.90	575.00 ± 52.96	544.05 ± 46.89	0.37	0.67
18–21d	18d BW (kg)	15.36 ± 1.10	16.84 ± 1.02	16.15 ± 0.92	0.51	0.63
	Final BW (kg)	16.53 ± 1.14	17.42 ± 1.29	16.91 ± 1.07	0.56	0.73
	ADG (g/d)	397.22 ± 27.69	300.00 ± 85.80	336.67 ± 39.23	0.27	0.70

Data are presented as mean ± SEM, *n* = 6 for each group.

a *P*_t_: Control vs PQ.

b *P*_t_: EA vs PQ.

**Figure 2:**
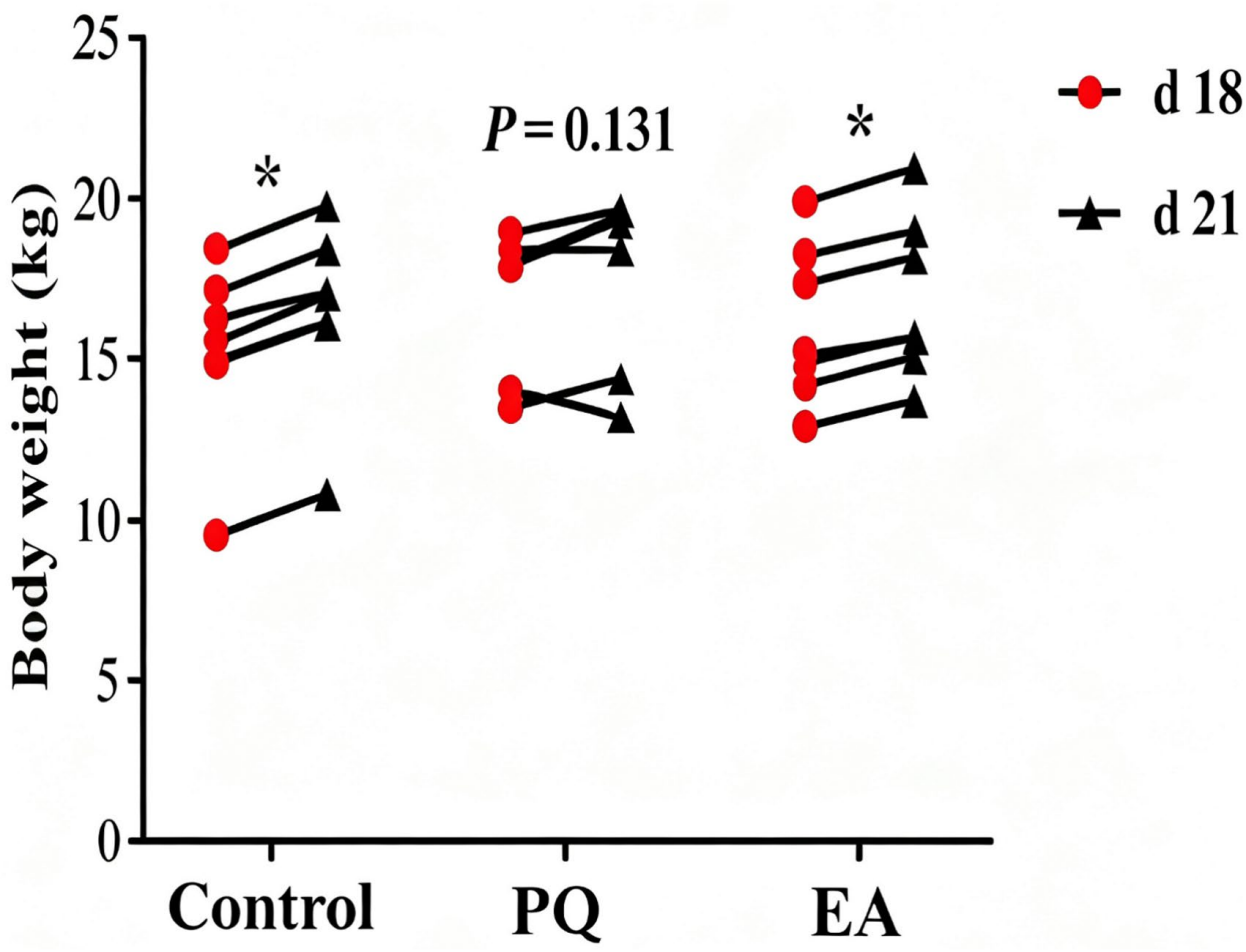
Changes in body weight from day 18 to day 21 in the control, PQ, and EA groups. Body weights on days 18 (d18) and 21 (d21) within each group were compared using a paired-samples *t*-test. *n* = 6 for each group. ^*^Indicates significant difference (*p* < 0.05).

### 3.2 Effects of EA supplementation on serum biochemical parameters in PQ-challenged piglets

As shown in Table 4, compared with the control group, PQ challenge significantly decreased serum ALB concentration (*p* < 0.01) and tended to decrease LDL concentration (*p* = 0.07). EA supplementation did not significantly alter any of the measured serum biochemical parameters relative to the PQ group (*p* > 0.05).

**Table 4:** Effects of EA supplementation on serum biochemical parameters in PQ-challenged piglets.

Item	Control group	PQ group	EA group	*P*_t_-value^a^	*P*_t_-value^b^
TP (g/L)	66.79 ± 2.75	64.68 ± 2.66	67.73 ± 2.28	0.60	0.95
ALB (g/L)	43.01 ± 0.93	37.22 ± 1.04	42.03 ± 1.50	< 0.01	0.27
GLB (g/L)	23.77 ± 2.63	27.47 ± 2.23	25.70 ± 2.14	0.24	0.93
ALT (U/L)	67.60 ± 10.26	68.47 ± 8.87	76.11 ± 8.48	0.95	0.39
AST(U/L)	168.57 ± 19.50	176.50 ± 21.56	199.00 ± 32.19	0.79	0.86
ALP (U/L)	206.86 ± 22.91	206.50 ± 21.21	195.71 ± 33.38	0.99	0.64
CREA (μmol/L)	91.86 ± 8.92	97.83 ± 3.34	97.43 ± 6.39	0.55	0.51
GLU (mmol/L)	8.31 ± 1.11	7.58 ± 0.31	7.91 ± 0.32	0.57	0.28
TG (mmol/L)	0.68 ± 0.05	0.68 ± 0.08	0.61 ± 0.06	0.96	0.18
CHO (mmol/L)	3.36 ± 0.30	2.73 ± 0.18	2.77 ± 0.17	0.11	0.78
HDL (mmol/L)	1.22 ± 0.14	1.12 ± 0.06	1.13 ± 0.10	0.52	0.59
LDL (mmol/L)	2.05 ± 0.20	1.57 ± 0.13	1.66 ± 0.12	0.07	0.88

Data are presented as mean ± SEM, *n* = 6 for each group.

a P_t_: Control vs. PQ.

b P_t_: EA vs PQ.

### 3.3 Effects of EA supplementation on antioxidant status in serum and longissimus dorsi muscle of PQ-challenged piglets

As shown in Figure. 3, PQ challenge significantly decreased T-AOC and GPx, as well as longissimus dorsi muscle SOD and GSH, compared with the control group (*p* < 0.05). EA supplementation significantly increased all four antioxidant-related indices relative to the PQ group (*p* < 0.05), indicating a partial restoration of antioxidant status in both serum and longissimus dorsi muscle.

**Figure 3:**
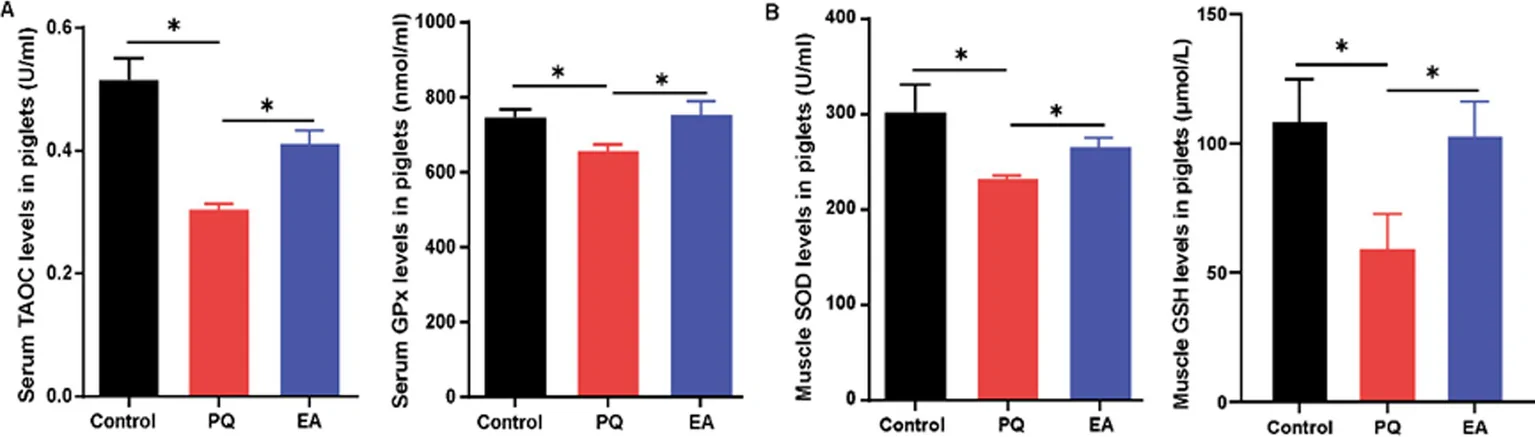
Effects of EA supplementation on antioxidant parameters in serum and *longissimus dorsi* muscle of PQ-challenged piglets. **(A)** Serum of T-AOC and GPx activity. **(B)** SOD activity and GSH content in the *longissimus dorsi* muscle. Data are presented as mean ± SEM. *n* = 6 for each group. ^*^Indicates significant difference (*p* < 0.05).

### 3.4 Effects of EA supplementation on longissimus dorsi muscle development and injury related gene expression in PQ-challenged piglets

As shown in Figure. 4, compared with the control group, PQ challenge significantly decreased the longissimus dorsi muscle mRNA abundance of *MYOD*, *MYOG*, *DESMIN*, and *CAV3* (*p* < 0.05), whereas *FOXO1*, *P21*, and *MCP-1* were not significantly altered (*p* > 0.05). Relative to the PQ group, EA supplementation significantly increased *FOXO1*, *P21*, *CAV3*, and *MCP-1* mRNA abundance (*p* < 0.05), while *MYOD*, *MYOG*, and *DESMIN* remained unchanged (*p* > 0.05).

**Figure 4:**
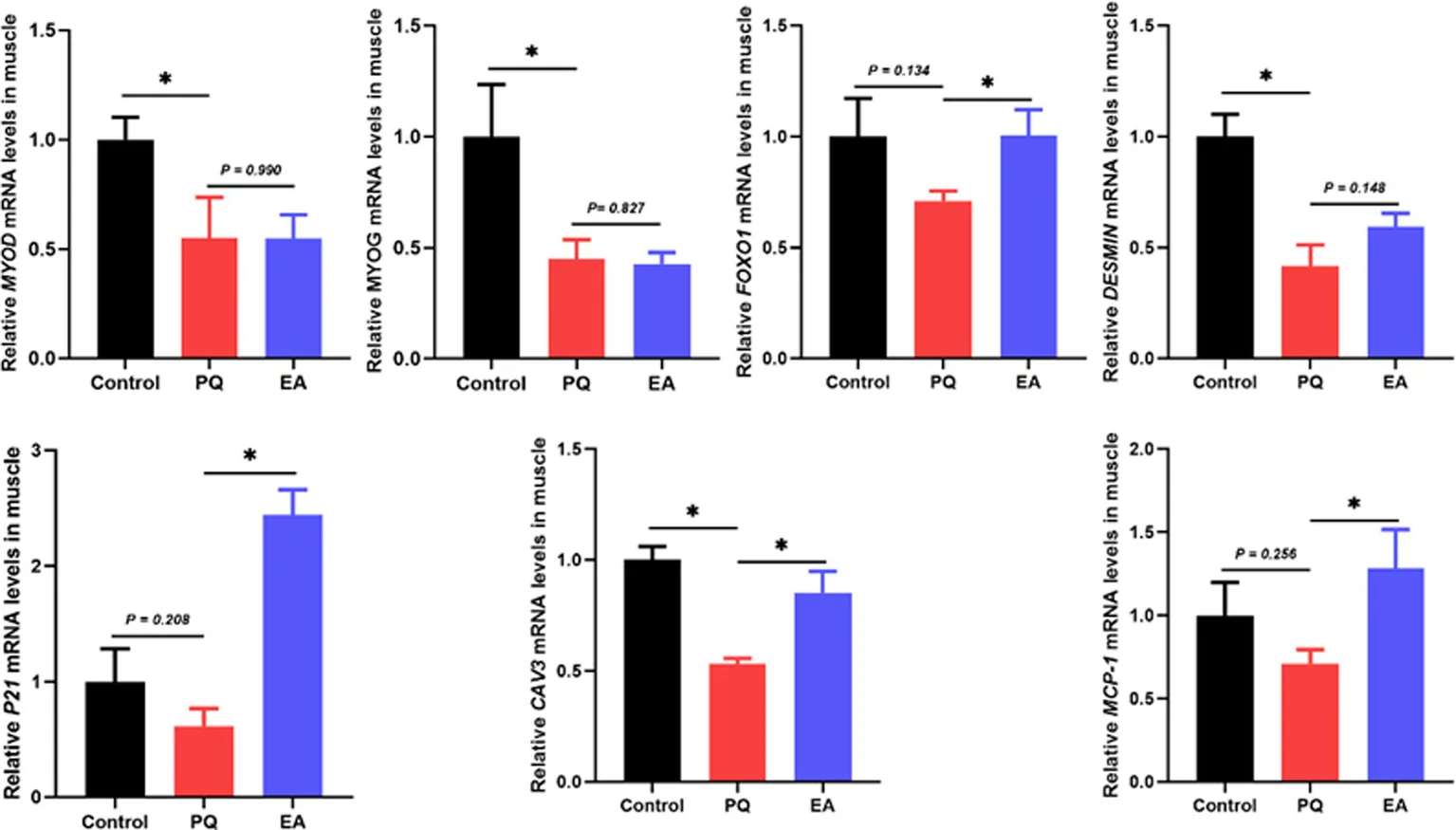
Effects of EA supplementation on muscle development and injury-related gene expression in the longissimus dorsi muscle of PQ-challenged piglets. Relative mRNA expression levels of *MYOD*, *MYOG*, *FOXO1*, *DESMIN*, *P21*, *CAV3*, and *MCP-1* were determined by RT-qPCR. Data are presented as mean ± SEM. *n* = 6 for each group. ^*^Indicates significant difference (*p* < 0.05).

### 3.5 Effects of EA supplementation on serum and longissimus dorsi muscle amino acid profiles in PQ-challenged piglets

As shown in Table 5, PQ challenge altered the serum amino acid profile. Compared with the control group, serum Asp., Ser, Gly, Ile, and Phe concentrations were significantly decreased (*p* < 0.05), while Ala and Pro showed decreasing trends (0.05 ≤ *p* < 0.10). Relative to the PQ group, EA supplementation significantly increased serum Asp concentration (*p* < 0.05), and Cys showed an increasing trend (*p* = 0.06); no other serum amino acids were significantly affected.

**Table 5:** Effects of EA supplementation on serum amino acid contents in PQ-challenged piglets (μg/mL).

Item	Control group	PQ group	EA group	Pt-value^a^	Pt-value^b^
Tau	3.39 ± 0.79	2.13 ± 0.26	2.60 ± 0.14	0.18	0.16
Asp	0.62 ± 0.13	0.23 ± 0.22	0.48 ± 0.05	0.03	0.02
Thr	4.50 ± 0.74	3.51 ± 0.54	3.05 ± 0.20	0.31	0.47
Ser	6.43 ± 0.46	4.72 ± 0.24	5.09 ± 0.31	0.01	0.36
Glu	17.60 ± 1.62	15.28 ± 2.59	14.20 ± 1.40	0.47	0.66
Gly	26.53 ± 1.60	18.95 ± 2.31	19.68 ± 1.64	<0.01	0.57
Ala	24.65 ± 3.38	17.35 ± 1.60	19.69 ± 2.19	0.09	0.41
Cit	4.37 ± 0.27	3.74 ± 0.53	3.51 ± 0.36	0.31	0.73
Val	9.90 ± 0.81	7.91 ± 0.73	7.65 ± 0.39	0.10	0.76
Cys	0.78 ± 0.28	0.31 ± 0.08	0.57 ± 0.70	0.14	0.06
Met	1.66 ± 0.19	1.38 ± 0.19	1.24 ± 0.86	0.33	0.51
Ile	5.74 ± 0.56	3.54 ± 0.76	3.51 ± 0.31	0.02	0.97
Leu	8.50 ± 0.70	6.92 ± 0.67	6.31 ± 0.29	0.14	0.44
Tyr	3.69 ± 0.34	2.76 ± 0.47	2.44 ± 0.18	0.14	0.53
Phe	5.00 ± 0.47	3.56 ± 0.35	4.03 ± 0.24	0.03	0.15
Orn	4.74 ± 0.41	4.45 ± 0.60	4.45 ± 0.83	0.70	0.99
Lys	11.94 ± 1.07	10.16 ± 1.64	7.93 ± 0.68	0.38	0.25
His	3.35 ± 0.25	2.85 ± 0.46	2.88 ± 0.33	0.36	0.23
Arg	9.01 ± 1.55	5.52 ± 1.66	5.53 ± 1.12	0.16	0.89
Pro	7.91 ± 0.54	6.14 ± 0.67	6.54 ± 0.72	0.07	0.70

Data are presented as mean ± SEM, *n* = 6 for each group.

a P_t_: Control vs. PQ.

b P_t_: EA vs. PQ.

As shown in Table 6, In longissimus dorsi muscle, PQ challenge significantly decreased Tau, Glu, Cys, and Pro concentrations and increased Thr, Orn, and Lys concentrations compared with the control group (*p* < 0.05), with Gly showing a decreasing trend (*p* = 0.08). Compared with the PQ group, EA supplementation significantly increased Tau, Glu, Ile, Leu, Phe, and Pro concentrations and decreased Thr, Orn, and Lys concentrations (*p* < 0.05). Collectively, EA exerted a broader effect on the muscle amino acid profile than on the serum amino acid profile.

**Table 6:** Effects of EA supplementation on longissimus dorsi muscle amino acid contents in PQ-challenged piglets (μg/mL).

Item	Control group	PQ group	EA group	Pt-value^a^	Pt-value^b^
Tau	643.44 ± 10.93	461.26 ± 55.12	885.30 ± 144.57	0.02	0.04
Asp	18.17 ± 1.27	29.00 ± 10.43	34.13 ± 5.22	0.35	0.67
Thr	67.89 ± 1.41	93.33 ± 6.60	57.94 ± 6.21	0.01	<0.01
Ser	117.03 ± 2.31	98.42 ± 10.81	99.48 ± 9.08	0.15	0.94
Glu	246.81 ± 3.33	31.50 ± 5.63	260.95 ± 34.28	<0.01	<0.01
Gly	694.16 ± 11.35	572.19 ± 67.01	591.80 ± 83.04	0.08	0.86
Ala	672.90 ± 10.34	608.40 ± 61.83	661.70 ± 112.74	0.35	0.69
Cit	58.77 ± 1.12	48.14 ± 6.72	49.74 ± 8.15	0.18	0.88
Val	47.85 ± 0.79	50.20 ± 4.58	50.69 ± 5.49	0.63	0.95
Cys	31.68 ± 1.28	12.60 ± 5.92	19.37 ± 6.26	0.02	0.45
Met	27.35 ± 1.26	30.53 ± 1.58	35.95 ± 5.44	0.14	0.33
Ile	54.21 ± 2.08	49.00 ± 4.69	64.17 ± 8.98	0.31	0.02
Leu	88.36 ± 1.88	84.24 ± 6.52	108.18 ± 10.29	0.57	0.03
Tyr	25.30 ± 3.86	31.37 ± 6.80	35.73 ± 7.30	0.44	0.66
Phe	56.84 ± 3.50	48.29 ± 4.13	65.64 ± 5.93	0.14	0.04
Orn	5.42 ± 2.47	28.59 ± 6.32	13.77 ± 2.42	0.01	0.04
Lys	109.38 ± 2.79	195.39 ± 17.94	110.06 ± 27.07	<0.01	0.02
His	5.42 ± 3.27	10.57 ± 3.62	12.28 ± 7.12	0.31	0.83
Arg	7.15 ± 4.13	5.14 ± 1.70	8.43 ± 2.46	0.67	0.29
Pro	150.53 ± 11.28	10.93 ± 4.69	84.25 ± 18.39	<0.01	<0.01

Data are presented as mean ± SEM, *n* = 6 for each group.

a Pt: Control vs. PQ.

b Pt: EA vs. PQ.

### 3.6 Effects of EA supplementation on amino acid transport- and metabolism-related gene expression in longissimus dorsi muscle

As shown in Figure.5, compared with the control group, PQ challenge significantly decreased the mRNA abundance of S*LC1A1*, *SLC6A6*, *SLC25A2*, and *SLC36A1* and increased *ODC* expression in longissimus dorsi muscle (*p* < 0.05). *PRODH* expression also showed an increasing trend following PQ challenge (*p* = 0.052). Relative to the PQ group, EA supplementation significantly increased *SLC1A1* and *SLC6A6* expression and decreased *PRODH*, *OAT*, and *ODC* expression (*p* < 0.05). EA supplementation also tended to decrease *SLC1A2* expression (*p* = 0.096), whereas *SLC25A2* and *SLC36A1* were not significantly altered (*p* > 0.05).

**Figure 5:**
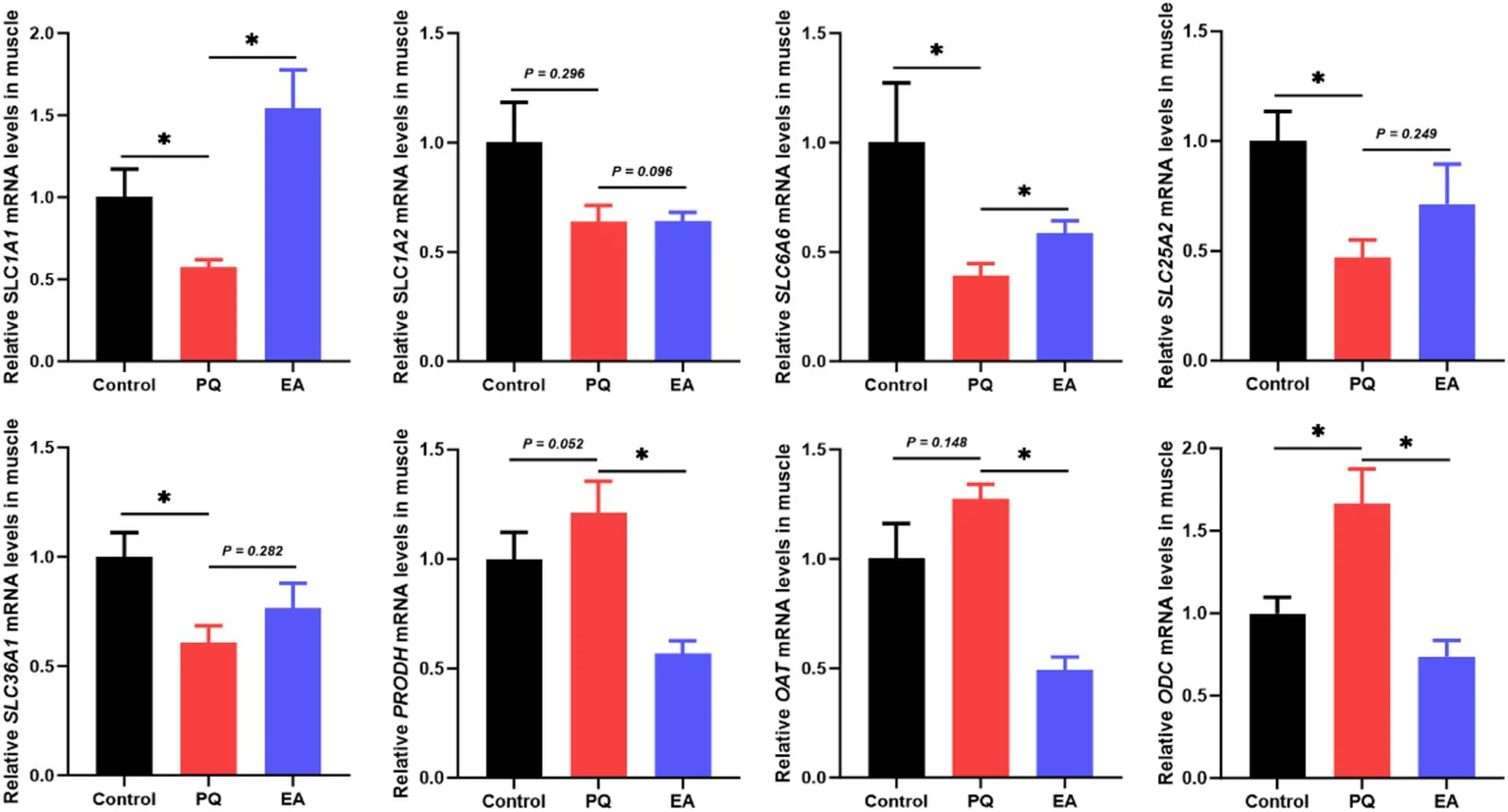
Effects of EA supplementation on amino acid transport and metabolism related gene expression in the longissimus dorsi muscle of PQ-challenged piglets. Relative mRNA expression levels of *SLC1A1, SLC1A2, SLC6A6, SLC25A2, SLC36A1, PRODH, OAT,* and *ODC* were determined by RT-qPCR. Data are presented as mean ± SEM. *n* = 6 for each group. ^*^Indicates significant difference (*p* < 0.05).

### 3.7 Effects of EA supplementation on the longissimus dorsi muscle long-chain fatty acid profile in PQ-challenged piglets

As shown in Table 7, compared with the control group, PQ challenge significantly increased longissimus dorsi muscle C18:3N-6, C20:1, C20:3N-6, and C24:0 concentrations, while decreasing C18:3N-3 and C22:1N-9 concentrations (*p* < 0.05). PQ challenge also tended to decrease C16:1, C18:1N-9T, and C20:2 and to increase C20:4N-6 and C22:6N-3 (0.05 ≤ *p* < 0.10). Relative to the PQ group, EA supplementation significantly increased C18:3N-3 and C22:1N-9 concentrations (*p* < 0.05), tended to increase C16:1, and tended to decrease C18:0 and C20:3N-6 (0.05 ≤ *p* < 0.10). Thus, EA supplementation partially normalized selected PQ-associated changes in the longissimus dorsi muscle fatty acid profile.

**Table 7:** Effects of EA supplementation on the longissimus dorsi muscle long-chain fatty acid profile in PQ-challenged piglets.

Item	Control group	PQ group	EA group	Pt-value^a^	Pt-value^b^
C14:0	12.09 ± 0.29	13.67± 0.99	12.03 ± 0.66	0.158	0.186
C16:0	241.48 ± 5.49	226.10 ± 14.26	208.85 ± 5.68	0.465	0.259
C16:1	28.37± 0.99	22.64 ± 2.45	28.16 ± 1.54	0.055	0.075
C17:0	2.22 ± 0.06	2.19 ± 0.35	2.00 ± 1.48	0.806	0.397
C18:0	112.70 ± 2.83	138.99 ± 14.38	113.61± 2.56	0.103	0.087
C18:1N9T	1.12 ± 0.05	0.97 ± 0.06	0.99± 0.05	0.099	0.739
C18:1N9C	260.11 ± 7.95	279.83 ± 24.96	269.85 ±8.67	0.469	0.695
C18:2N6C	259.71 ± 7.75	219.48 ± 24.25	250.33±9.36	0.145	0.234
C20:0	1.43 ± 0.07	1.55 ± 0.06	1.42 ± 0.08	0.616	0.560
C18:3N6	1.28± 0.07	1.55 ± 0.57	1.46± 0.12	0.012	0.528
C20:1	1.22 ± 0.14	2.85 ± 0.51	2.15 ± 0.11	0.011	0.171
C18:3N3	19.28± 0.44	13.23 ± 1.22	17.02 ± 0.84	<0.01	0.024
C20:2	8.33±0.30	7.05±0.57	8.05±0.34	0.078	0.149
C20:3N6	5.44±0.14	6.96±0.64	5.68±0.15	0.043	0.060
C22:1N9	2.55±0.11	2.04±0.17	2.38±0.12	0.004	0.046
C20:4N6	54.83±1.25	66.21±5.15	59.22±3.44	0.057	0.271
C24:0	7.55±0.22	9.82±0.94	8.37±0.33	0.040	0.148
C22:6N3	12.38±0.29	15.54±1.48	14.90±0.59	0.063	0.681

Data are presented as mean ± SEM, *n* = 6 for each group.

a P_t_: Control vs. PQ.

b P_t_: EA vs. PQ.

### 3.8 Effects of EA supplementation on fatty acid transport- and metabolism-related gene expression in longissimus dorsi muscle

As shown in Figure 6, compared with the control group, PQ challenge significantly decreased the longissimus dorsi muscle mRNA abundance of *FABP3*, *FABP4*, *CD36*, *DGAT1*, *PLIN3*, and *CPT1B* (*p* < 0.05). In addition, *FABP1*, *CAV1*, and *PLIN2* showed decreasing trends, whereas *FATP4*, *PNPLA2*, and *PPARA* were not significantly altered (*p* > 0.05). Compared with the PQ group, EA supplementation significantly increased *FABP3*, *CD36*, and *PLIN3* mRNA abundance (*p* < 0.05) and tended to increase *CPT1B* expression (*p* = 0.090). No significant differences were observed in *FABP1*, *FABP4*, *FATP4*, *CAV1*, *DGAT1*, *PLIN2*, *PNPLA2*, or *PPARA* expression between the PQ and EA groups (*p* > 0.05). These data show that the transcriptional response to EA was most evident for genes related to fatty acid handling and lipid-droplet regulation.

**Figure 6:**
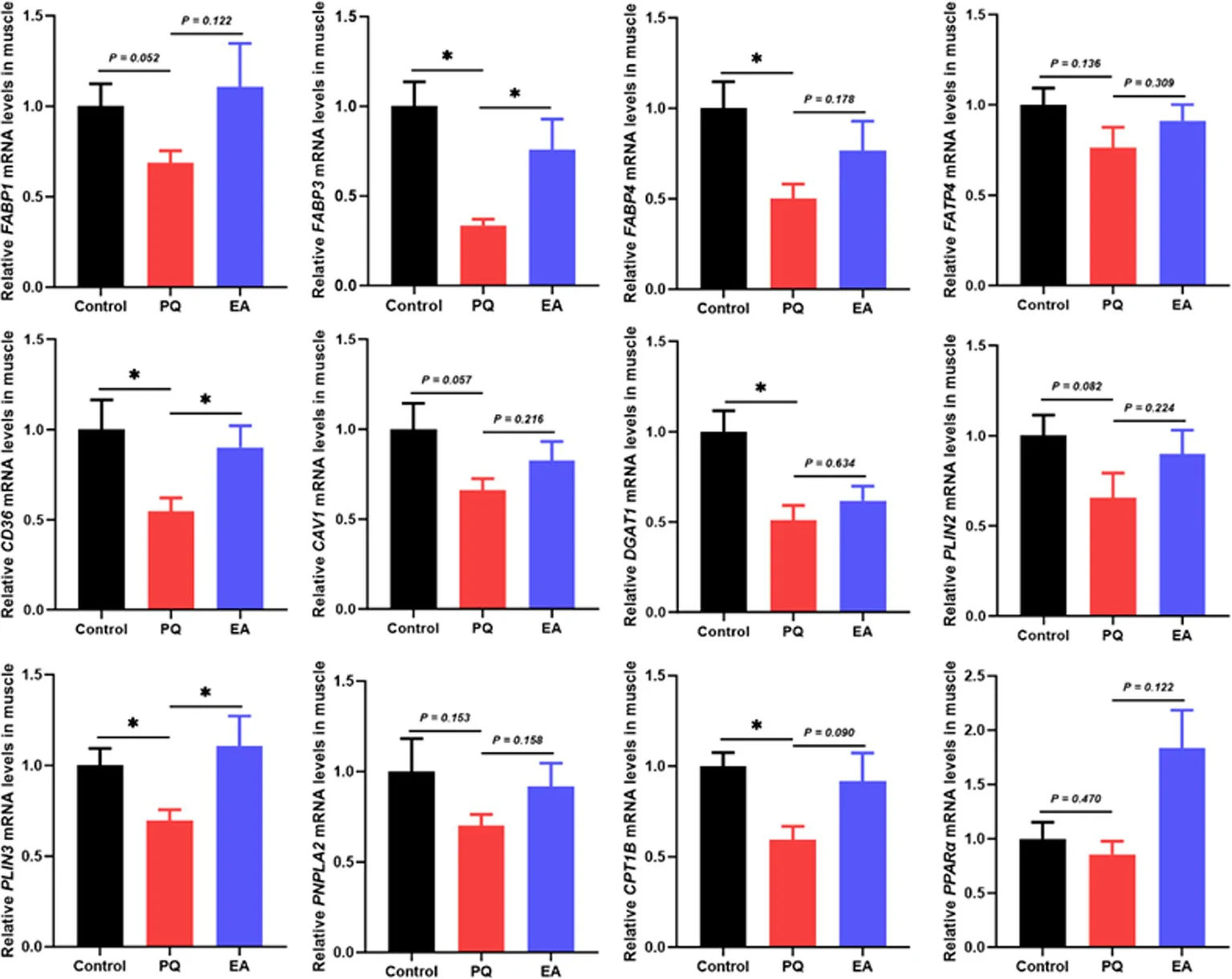
Effects of EA supplementation on fatty acid transport and metabolism related gene expression in the longissimus dorsi muscle of PQ-challenged piglets. Relative mRNA expression levels of *FABP1, FABP3, FABP4, FATP4, CD36, CAV1, DGAT1, PLIN2, PLIN3, PNPLA2, CPT1B*, and *PPARα* were determined by RT-qPCR. Data are presented as mean ± SEM. *n* = 6 for each group. ^*^Indicates significant difference (*p* < 0.05).

## 4 Discussion

The present study demonstrated that PQ-induced OS markedly disrupted antioxidant capacity and nutrient metabolism in the longissimus dorsi muscle of piglets, whereas dietary EA supplementation partially alleviated these alterations. Although EA supplementation did not significantly improve overall growth performance during the experimental period, clear protective effects were observed at the biochemical and molecular levels, particularly in antioxidant defense, amino acid homeostasis, lipid metabolism, and muscle development. These findings suggest that the protective effects of EA on muscle may occur before substantial improvements in whole-body growth become evident.

OS is characterized by excessive production of reactive oxygen species and an imbalance between oxidant production and antioxidant defense, which can impair cellular macromolecules, mitochondrial function, and tissue metabolism (1, 3). PQ is a potent redox-cycling agent that generates reactive oxygen species and has been widely used to induce oxidative injury (30). Previous studies in piglets have shown that PQ challenge causes intestinal and hepatic oxidative damage, whereas EA supplementation enhances antioxidant capacity and alleviates tissue injury (5–7). Consistent with these observations, the present study showed that PQ challenge impaired antioxidant status in the longissimus dorsi muscle, while EA supplementation partially restored antioxidant-related parameters. EA possesses strong antioxidant properties and can modulate endogenous antioxidant defense systems, including Nrf2-associated signaling (5, 8, 10). Therefore, improvement of muscle redox homeostasis may represent an important mechanism through which EA protects skeletal muscle metabolism during OS.

EA influenced muscle-related gene expression in a marker-specific manner. Oxidative stress can interfere with myogenic differentiation (31, 32), consistent with the PQ-associated reductions in *MYOD*, *MYOG*, *DESMIN*, and *CAV3* expression. Among these markers, EA significantly increased *CAV3* expression relative to PQ, whereas *MYOD*, *MYOG*, and *DESMIN* were not significantly altered. Given the role of *CAV3* in muscle membrane organization, its upregulation suggests a potential contribution to the maintenance of muscle cellular integrity. This selective response distinguishes the effect on *CAV3* from the absence of a detectable response in the other three markers.

The increases in *FOXO1*, *P21*, and *MCP-1* expression following EA supplementation point to additional effects on muscle regulatory processes. *FOXO1* is involved in muscle metabolic regulation, although muscle-specific overexpression has also been associated with reduced muscle mass in mice (33). Its increase should therefore be considered a regulatory response whose significance depends on protein activity and downstream targets. *P21* coordinates cell-cycle withdrawal during myogenic differentiation (34), suggesting that its elevation may relate to cell-cycle regulation. *MCP-1*/*CCL2* participates in monocyte/macrophage recruitment during muscle repair (35); increased expression may reflect modulation of inflammatory or repair-related signaling. Because *FOXO1*, *P21*, and *MCP-1* were not significantly changed by PQ versus control, their responses to EA represent additional transcriptional effects rather than demonstrated reversal of PQ-induced alterations. These findings identify candidate processes for mechanistic investigation without equating all three responses with enhanced myogenesis.

The amino acid findings suggest that EA modulates the intramuscular nutrient pool during oxidative challenge. Relative to PQ, EA increased muscle Tau, Glu, Ile, Leu, Phe, and Pro and decreased Thr, Orn, and Lys, whereas serum changes were largely confined to increased Asp. The parallel increases in muscle Tau and SLC6A6 expression are particularly relevant because taurine supports normal muscle function (36, 37). EA also increased SLC1A1 and decreased PRODH, OAT, and ODC expression, indicating coordinated changes in selected transport- and metabolism-related transcripts; SLC25A2 and SLC36A1 were not significantly affected. Increased muscle leucine is biologically relevant given its established role in stimulating protein synthesis (19, 20). Together, these observations suggest that altered amino acid availability and handling may contribute to the metabolic response to EA, while direct measurements of transport and protein turnover would clarify the underlying processes.

EA also affected genes involved in fatty acid handling. The increases in FABP3, CD36, and PLIN3 relative to PQ are consistent with modulation of intracellular fatty acid transport, cellular uptake, and lipid-droplet regulation, respectively (26, 27, 29). These processes connect lipid availability with storage and utilization and may contribute to the muscle metabolic effects of EA. The response was selective: FABP4 and DGAT1 were not significantly altered by EA, and CPT1B showed an upward trend that did not reach significance. Thus, the transcriptional evidence most directly supports effects on fatty acid handling and lipid-droplet regulation.

At the metabolite level, EA increased muscle C18:3 N-3 and C22:1 N-9 relative to PQ, counteracting the decreases associated with PQ challenge. Changes in C16:1, C18:0, and C20:3 N-6 did not reach statistical significance. The significant responses of C18:3 N-3 and C22:1 N-9 complement the increases in FABP3, CD36, and PLIN3 and support an effect of EA on selected aspects of muscle lipid metabolism. This interpretation is consistent with evidence that nutritional and environmental conditions influence porcine muscle fatty acid composition (29, 38).

Several limitations should be acknowledged. Only one EA dose (100 mg/kg diet) was evaluated, leaving its dose dependence and optimal supplementation level unresolved. Muscle morphology and function were not assessed, and the small sample size and short post-challenge period limited evaluation of longer-term outcomes. In addition, metabolite concentrations and mRNA abundance provide indirect evidence of metabolic regulation. Future studies should compare multiple EA doses and incorporate muscle fiber morphology, contractile function, and longer follow-up. Protein-level analyses and measurements of metabolic flux would further clarify how the antioxidant and metabolic responses relate to muscle structure and function.

## 5 Conclusion

In conclusion, dietary EA supplementation improved antioxidant status in PQ-challenged piglets, as reflected by increased serum T-AOC and GPx activity and muscle SOD activity and GSH content. EA also modulated muscle amino acid profiles, increased C18:3 N-3 and C22:1 N-9, and upregulated *SLC1A1, SLC6A6, FABP3, CD36*, and *PLIN3* relative to PQ. These findings support the potential of EA to alleviate selected biochemical and metabolic disturbances associated with oxidative challenge. Although growth performance was not significantly improved during the experimental period, the antioxidant and metabolic responses provide a basis for further evaluation of EA as a nutritional approach to supporting muscle homeostasis under oxidative stress.

## Data Availability

The raw data supporting the conclusions of this article will be made available by the authors, without undue reservation.
